# A Carbon-Based Nanomaterial with Dichotomous Effects: Antineoplastic on Oral Cancer Cells and Osteoinductive/Chondroinductive on Dental Pulp Stem Cells

**DOI:** 10.3390/jfb16030109

**Published:** 2025-03-19

**Authors:** Milica Jaksic Karisik, Nataša Jović Orsini, Jelena Carkic, Milos Lazarevic, Dijana Mitić, Bojan Jokanovic, Vukoman Jokanović, Jelena Milasin

**Affiliations:** 1 School of Dental Medicine, University of Belgrade, 11000 Belgrade, Serbia; milica.jaksic@stomf.bg.ac.rs (M.J.K.); jelena.carkic@stomf.bg.ac.rs (J.C.); milos.lazarevic@stomf.bg.ac.rs (M.L.); dijana.trisic@stomf.bg.ac.rs (D.M.); 2 Vinča Institute of Nuclear Sciences—National Institute of the Republic of Serbia, University of Belgrade, P.O. Box 522, 11001 Belgrade, Serbia; natasaj@vin.bg.ac.rs; 3 SGL Carbon, 86405 Meitingen, Bavaria, Germany; bojan.jokanovic@yahoo.de; 4 Innovative Company ALBOS DOO, 11000 Belgrade, Serbia; vjokan@gmail.com

**Keywords:** oral cancer, carbon nanomaterial, CBN/PVP, milled graphite, cancer therapy, tissue regeneration, osteogenesis, chondrogenesis

## Abstract

Background: Oral cancer is an aggressive malignancy with modest survival rates. It also causes disfigurement following surgical removal of the tumor, thus highlighting the need for new cancer treatment and tissue repair modalities. Carbon-based nanomaterials have emerged as promising tools in both anticancer and regenerative therapies. Objectives: We aimed to synthesize a new carbon-based nanomaterial (CBN) and test its antineoplastic effects, as well as its potential regenerative capacity. Materials and Methods: A carbon nanomaterial, obtained by ball milling graphite flakes, was functionalized with polyvinylpyrrolidone (CBN/PVP). Its physicochemical properties were explored with X-ray diffraction (XRD), attenuated total reflection–Fourier transform infrared spectroscopy (ATR-FTIR), micro-Raman spectroscopy, fluorescent and scanning electron microscopy, and wettability analysis. For the antineoplastic effects investigation, oral cancer cells were treated with CBN/PVP and examined with MTT and migration assays, as well as cell-cycle and ROS production analyses. Gene expression was determined by qPCR. To examine the pro-regenerative capacity of CBN/PVP, dental pulp stem cell cultures (DPSCs) were treated with the nanomaterial and subjected to osteo- and chondro-induction. Results: Lower concentrations of CBN/PVP (50, 100 μg/mL) applied on cancer cells exerted remarkable cytotoxic effects, induced G1 cell-cycle arrest, and reduced cancer cell invasion potential by different mechanisms, including downregulation of the PI3K/AKT/mTOR pathway. In contrast, the addition of 50 µg/mL of CBN/PVP to DPSCs stimulated their survival and proliferation. CBN/PVP significantly enhanced both the osteogenic (*p* < 0.05) and chondrogenic (*p* < 0.01) induction of DPSCs. Conclusions: The novel carbon-based nanomaterial displays unique characteristics, making it suitable in anticancer and regenerative therapies concomitantly.

## 1. Introduction

Oral cancer is one of the most prevalent malignancies, causing about 177,000 deaths each year [1]. Oral squamous cell carcinoma (OSCC) is the most frequent oral cancer, accounting for more than 90% of all oral neoplasms. Despite significant therapeutic advancements in recent decades, the 5-year disease-free survival rate remains at only about 50% [2], and in the future, OSCC incidence is likely to become even higher, with an estimated 856,000 new cases worldwide per year by 2035 [3]. OSCCs are aggressive neoplasms prone to relapses and metastases, even after effective surgical therapy, chemotherapy, and radiation treatment [4]. These cancers are usually diagnosed late, at an untreatable stage, when malignant cells have become intrinsically drug-resistant. Chemoresistance and the serious systemic adverse effects of current therapeutic modalities highlight the need for novel approaches to overcome these limitations, especially in the fields of drug delivery and tumor targeting [5]. Not only is OSCC an aggressive malignancy, but the surgical removal of the tumor often leaves severe sequelae and requires reconstructive/regenerative procedures.

Over the past decade, nanomaterials have emerged as a promising tool both in both cancer diagnosis and treatment [6]. Nanomaterials are chemical substances with a particle size below 100 nm; they are composed of different materials (organic, inorganic, or hybrid), and are structured in various shapes (tube, wires, ribbons, needle, sphere, capsule, rod, etc.) and dimensions (zero-dimensional, 1D, 2D, or 3D). These materials possess great potential in overcoming some of the conventional limitations in cancer therapy, such as the rapid metabolism and elimination of drugs, failure to attain the desired target site concentration, non-specific cytotoxicity, and others [7].

Among others, carbon nanostructures such as graphene, carbon nanotubes, multi-layered graphitic sheets, carbon dots, nanodiamonds, graphene oxide, and graphite have been widely studied for their potential applications in biomedicine. These materials have been explored for targeted drug and protein delivery to specific cancer cell lines, noninvasive and highly sensitive diagnostics, and biosensors. However, biocompatibility and biodegradability are crucial factors for the biomedical application of nano-objects. Therefore, the most important step is the functionalization of carbon nanomaterials [8]. Functionalizing the surface of carbon nanostructures with oxygen- or nitrogen-based functional groups modifies their surface properties, enhances their wettability in aqueous media, and facilitates interactions with biological entities such as cells and tissues. Very precise control of the surface wettability of a carbon nanomaterial is particularly important for cell adhesion on its surface, influencing the interactions between cells and the carbon-based nanomaterial. It is known that hydrophilic surfaces are favorable for short-term cell adhesion and proliferation for many cells, like fibroblasts, endothelial cells, osteoblastic cells, etc. [9,10]. Carbon-based nanomaterials can also be functionalized with different polymers, such as polyethylene glycol (PEG) and polyethylenimine (PEI). In this study, we used polyvinylpyrrolidone (PVP), a water-soluble, non-toxic polymer surfactant synthesized via the polymerization of the N-vinyl pyrrolidone monomer. PVP has good water-solubility, biocompatibility, and biodegradability. It is a non-ionic, temperature-resistant, and Ph-stable polymer approved by the US Food and Drug Administration (FDA) as a food additive. As it possesses an amphiphilic structure consisting of a hydrophilic pyrrolidone moiety and hydrophobic alkyl groups, PVP has also been used as a capping agent to improve the stability and dispersibility of graphite derivatives in aqueous and organic solvents, as well as their biocompatibility toward biological systems. It functions as a surface stabilizer, nanoparticle dispersant, and reduced agent.

Previous studies have investigated the potential of some carbon-based materials, such as graphene oxide (GO), for instance, as nanocarriers for various molecules in the management of OSCC, including anticancer drugs and micro-RNAs [6,11]. Ou et al. revealed that PEI-functionalized GO possesses low toxicity and the potential to be used as a carrier of miRNA in the treatment of OSCC. On the other hand, PVP-functionalized GO, due to its biocompatibility and immunoenhancement effects, could be used as an immunoadjuvant candidate [6,8].

The anticancer effects of carbon-based nanomaterials may be attributed to their ability to inhibit cancer-promoting pathways and genes while activating tumor suppressor genes. One of the most frequently upregulated signaling pathways in oral cancer is the PI3K/Akt/mTOR pathway, which regulates key cellular and metabolic processes in OSCC [12]. Due to its central role in tumor progression, this pathway is a promising target for carbon-based nanomaterials. Additionally, suppressing the anti-apoptotic *BCL-2* gene has been shown to significantly enhance apoptosis in cancer cells [13]. In contrast, the tumor suppressor PTEN counteracts cancer cell proliferation by inhibiting the PI3K/Akt pathway. Another signaling cascade frequently altered in oral cancer, and a potential therapeutic target, is the NOTCH1 pathway [14].

At the same time, thanks to their versatility and biocompatibility, in non-cancerous settings, i.e., normal cell settings, carbon-based nanomaterials possess the ability to promote cell growth, differentiation, and bone formation [15]. Consequently, these nanomaterials could be used not only as efficient anticancer agents, but as a promising material for tissue regeneration as well.

The aims of this study were as follows: (1) to synthesize and characterize a novel, carbon-based nanomaterial, obtained through the strong ball milling of pristine graphite and subsequently functionalized with polyvinylpyrrolidone (CBN/PVP), (2) to investigate this material’s possible antineoplastic effects on oral cancer cells, and (3) to examine its potential to induce the osteogenic and chondrogenic differentiation of normal DPSCs.

## 2. Materials and Methods

### 2.1. Synthesis and PVP Functionalization of the Carbon-Based Nanomaterial

Graphite flakes (with lateral sizes below 10 µm, SGL Carbon, Meitingen, Germany) were used to obtain the carbon-based nanomaterial through ball-milling processing. The mechanochemical treatment was completed in a planetary ball mill (Fritsch Pulverisette 5) with zirconium milling balls (∼10 mm in diameter) in an air atmosphere. The milling was performed for 2 h at a basic disc rotation speed of 320 rpm. The mass ratio of zirconium dioxide balls and graphite flakes was 1:20. After mechanical treatment, the obtained product was transferred to an autoclave and mixed with deionized water enriched with 3% H_2_O_2_ at a concentration of 1:100. The mixture was kept at 180 °C for 5 h. After cooling to room temperature, the obtained product was functionalized with PVP and used as a surface-active substance. The unreacted PVP molecules were removed from the mixture by washing with a large amount of hot water. The washed PVP-treated graphitic precipitate was subjected to filtration, using filter paper with a mesh diameter of 0.2 μm. Subsequently, the precipitate was removed from the paper, transferred to a ceramic dish, and dried at 45 °C in a vacuum oven.

### 2.2. Characterization of Pristine Graphite and CBN/PVP

Pristine and PVP-functionalized carbon-based nanoparticles were characterized using X-ray diffraction (XRD), as well as Fourier-transform infrared (FTIR) and micro-Raman spectroscopies. The XRD experiment was performed on a Rigaku SmartLab diffractometer (Tokyo, Japan) with Cu *Kα* (*λ_α_*_1_ = 1.54059 Å, *λ_α_*_2_ = 1.54441 Å) and *Kβ* (*λ_β_* = 1.39217 Å) radiation in a Bragg-Brentano geometry in the range of 10–60° 2*θ*, with a step of 0.02° and a speed counting rate of 2°/min. The FTIR spectra were collected in the spectral range of 4000 to 400 cm^−1^ using a Thermo Scientific Nicolet iS 50 spectrometer working in the ATR mode. The Raman spectrum of PVP-functionalized graphitic nanoparticles was collected using a DXR Raman microscope (Thermo Scientific, Waltham, MA, USA) in the spectral range 0–3300 cm^−1^ using a HeNe 532 nm gas laser working with a laser power of 2 mW.

Fluorescent microscopy (AxioImager A1, Zeiss, Berlin, Germany) and scanning electron microscopy (SEM, JEOL JSM-5300, Tokyo, Japan) were used to investigate the morphology and microstructure of the obtained graphite-based material. Prior to SEM, the material was washed with ethanol, which removed the PVP. The wettability measurements of the CBN/PVP sample were performed using a goniometer 500 supplied with a camera (U4 series, Berlin, Germany) and a blue LED lamp, under a controlled humidity of 70%. To evaluate the contact angle, each of the samples was wetted with five drops of deionized water and five drops of ethylene glycol.

### 2.3. OSCC and DPSC Cell Cultures

In this study, primary cell cultures were established from OSCC tissues of five patients; a commercially available oral cancer cell line SCC-25 (ATCC^®^, CRL–1628™, Manassas, VA, USA) was also used, but since it had the same behavior as the primary cultures, they were all merged into a unique OSCC group. Tumor tissues were obtained from the Clinic for Maxillofacial Surgery, School of Dental Medicine, University of Belgrade, Belgrade, Serbia. In addition, cultures of dental pulp stem cells (DPSCs) isolated from extracted third molars were also generated. Third-molar extractions were performed at the Clinic for Oral Surgery. All biological samples were obtained following the acquisition of signed informed consent from each patient. The study received approval from the Ethics Committee of the School of Dental Medicine (36/19).

Primary cell cultures were generated as previously described, and cells were cultured in Dulbecco’s Modified Eagle Medium (DMEM) supplemented with 10% fetal bovine serum (FBS) and 1% antibiotic–antimycotic solution (all from Thermo Fisher Scientific, Waltham, MA, USA). The tumor cell culture medium was supplemented with 400 ng/mL of hydrocortisone. Cells were maintained in a humidified atmosphere with 5% CO_2_ at 37 °C, and the culture medium was replenished every two to three days. Upon reaching 80% confluence, cells were passaged for subsequent assays. Cells of the third passage were used in the experiments [16].

### 2.4. 3-(4,5-Dimethylthiazol-2-yl)-2,5-Diphenyltetrazolium Bromide (MTT) Assay

To evaluate the cytotoxic effect of the nanomaterial, we used 5 primary tumor cell cultures and a commercial SCC-25 cell line. For the MTT assay, 1 × 10^4^ cells per well were seeded into a 96-well plate. After 24 h, cells were treated with 100 µL of complete medium containing CBN/PVP at concentrations of 50, 100, and 200 µg/mL. Cells were then incubated for 24, 48, and 72 h. The MTT assay was also applied for the monitoring of cell viability in the course of differentiation. Following each incubation period, the supernatant was removed, and 100 µL of dimethyl sulfoxide (DMSO) (Sigma-Aldrich Inc., Waltham, MA, USA) was added to dissolve the formazan precipitates by gentle shaking at 37 °C. Optical density (OD) was measured at 540 nm using an ELISA reader (RT-2100c, Rayto, Shenzhen, China). Cell viability (%) was calculated using the following formula:

Cell viability% = (mean absorbance of treated cells/mean absorbance of control cells) × 100%

### 2.5. Cell-Cycle Analysis

For cell-cycle analysis, 1 × 10^6^ SCC-25 cells per sample were seeded in a 6-well plate and treated with CBN/PVP over a period of 24 h. Cells were collected, washed with phosphate-buffered saline (PBS) by centrifugation at 1400 rpm for 6 min, washed one more time, and centrifuged again under the same conditions. The cell pellet was then resuspended in 300 µL of PBS, and 700 µL of 96% ice-cold ethanol was gradually added dropwise. Cells were incubated in this ethanol solution at 4 °C for 2 h to ensure fixation. Following this, cells were centrifuged at 1700 rpm for 6 min, the ethanol was discarded, and cells were resuspended in PBS. Another round of centrifugation was performed, and the pellet was resuspended in 500 µL of PBS. To degrade RNA, 7 µL of RNase A (100 µg/mL stock) was added, and cells were incubated at 37 °C for 15 min. Before analysis, propidium iodide (PI) was added at a final concentration of 50 µg/mL. The cell-cycle phase distribution was then assessed using a BD FACSMelody™ flow cytometer with BD FACSChorus™ software (version 3.0) [17].

### 2.6. Transwell Invasion Assay and Wound Healing Assay

For the transwell invasion assay, the upper chamber of each well was coated with Corning^®^ Matrigel^®^ Matrix before seeding 1 × 10^5^ SCC-25 cells per well. In the lower chamber, 500 µL of complete growth medium supplemented with 50, 100 μg/mL CBN/PVP was added, while the control chamber contained only the complete growth medium. The plates were incubated for 24 h to allow cell invasion through the matrix. After incubation, cells that had migrated to the bottom of the upper chamber were fixed with 90% ethanol for 30 min, followed by staining with 0.2% Crystal Violet for another 30 min. Images of the stained cells were captured using an inverted light microscope (Primovert Zeiss, Berlin, Germany).

The wound healing assay was used to evaluate the effect of CBN/PVP on the migration of DPSCs; a standardized scratch was made in a confluent cell monolayer and the rate of wound closure was measured over time using ImageJ software 1.48 version (NIH, Bethesda, MD, USA).

### 2.7. ROS Detection by Flow Cytometry

SCC25 cells were seeded at 1 × 10^6^ cells per well in six-well plates and incubated for 24 h. Three groups were prepared: (1) an experimental group treated with different concentrations (50, 100 μg/mL) of CBN/PVP, (2) a positive control treated with 100 µM hydrogen peroxide (H_2_O_2_) to induce ROS, and (3) a negative control (untreated). After 24 h, cells were washed with PBS and stained with 10 µM Diacetyldichlorofluorescein (DCFH-DA) in serum-free medium at 37 °C for 30 min. DCFH-DA detects reactive oxygen species (ROS) by converting into fluorescent dichlorofluorescein (DCF) upon oxidation. Following staining, cells were washed, trypsinized, centrifuged, and resuspended in cold PBS with 10% FBS. The BD FACSMelody™ flow cytometer with a 488 nm laser was used to measure DCF fluorescence, with low flow rates to ensure accuracy. The results were normalized to the untreated control for ROS quantification [18].

### 2.8. Nanoparticle Uptake Assay

Tumor cells SCC-25 (10^6^/mL) were plated in six-well plates for the flow cytometry analysis of CBN/PVP uptake. After 24 h without treatment, cells were treated with CBN/PVP at concentrations of 50 µg/mL and 100 µg/mL for 24 h. Post three PBS washes, cells were trypsinized, centrifuged, and resuspended in 1 mL cold PBS with 10% FBS. Analysis was performed using a BD FACSMelody™ flow cytometer (488 nm laser, FSC linear, SSC logarithmic, BD FACSChorus™ v1.3.3) at low flow rates, as previously described [17].

### 2.9. CBN/PVP Effects on DPSCs’ Differentiation Induction

Dental pulp stem cells (DPSCs) were plated in 24-well culture plates at a density of 1 × 10^5^ cells per well in complete growth medium. Upon reaching 80% confluence, the cells were treated with complete medium, complete medium supplemented with 50 µg/mL CBN/PVP, chondrogenic and osteogenic differentiation media (as controls), and chondrogenic and osteogenic differentiation media enriched with 50 µg/mL CBN/PVP for osteogenic and hondrogenic differentiation (StemMACS™, Miltenyi Biotec, Tokyo, Japan) and cultured under standard conditions (5% CO_2_ at 37°C) for 14 days, with media changes every three days. The cell viability was measured using an MTT assay, as described in Section 2.4.

Cells were washed twice with PBS and fixed with 4% paraformaldehyde for 30 min at room temperature. For differentiation, cells were stained with a 2% Alizarin Red S solution (Centrohem, Belgrade, Serbia) for osteogenesis or 0.5% Safranin O (Sigma-Aldrich, St. Louis, MI, USA) for chondrogenesis. The samples were incubated for 30 min and then washed with distilled water. For the quantification of the staining, cells were de-stained using 10% acetyl pyridinium chloride, and absorbance was measured at 450 nm using a microplate reader (RT-2100c, Rayto, Shenzhen, China).

### 2.10. Isolation of RNA and Reverse-Transcription Polymerase Chain Reaction (RT-PCR)

Total RNA was extracted from cells using TRIzol Reagent (Invitrogen, Thermo Fisher Scientific, Waltham, MA, USA), following the manufacturer’s protocol. RNA concentration was measured with a BioSpec–nano Microvolume UV–Vis Spectrophotometer (Shimadzu Scientific Instruments, Columbia, MD, USA). For cDNA synthesis, 2 µg of total RNA was used with oligo d(T) primers and the RevertAid First Strand cDNA Synthesis Kit (Thermo Fisher Scientific).

### 2.11. Quantitative Polymerase Chain Reaction (qPCR)

Real-time qPCR was performed with the SensiFAST SYBR Hi-ROX Kit (Bioline, London, UK), cDNA, specific primers, and water. The expression levels of phosphatidylinositol 3-kinase (*PI3K*), protein kinase B (*AKT*), the mammalian target of rapamycin (*mTOR*), neurogenic locus notch homolog protein 1 (*NOTCH1*), hairy and enhancer of split-1 (*HES1*), hairy/enhancer-of-split related with YRPW motif 1 *(HEY1),* catenin beta-1 (*CTNNB1*), phosphatase and tensin homolog (*PTEN*), *G1/S-specific Cyclin-D1* (*CCND1*), snail family transcriptional repressor 1 (*SNAIL*), and B-cell lymphoma 2 (*BCL-2*) were analyzed, with glyceraldehyde 3-phosphate dehydrogenase *(GAPDH)* used as the reference gene. Gene expression was calculated using the 2^−∆Ct^ method [19]. Primer sequences are provided in Table S1.

### 2.12. Statistical Analysis

Statistical analysis was conducted using GraphPad Prism version 9 (GraphPad Software, Inc., San Diego, CA, USA). The Kolmogorov–Smirnov test was initially used to evaluate the normality of data distribution. For comparisons, an ordinary one-way analysis of variance (ANOVA) with a single pooled variance was performed, followed by Tukey’s post hoc test for multiple-group comparisons. Data are expressed as mean ± SD, and statistical significance was defined as * *p* < 0.05, ** *p* < 0.01, *** *p* < 0.001, and **** *p* < 0.0001, with significance thresholds set at *p* < 0.05.

## 3. Results

### 3.1. Material’s Characteristics

#### 3.1.1. Raman Spectroscopy

**Figure 1:**
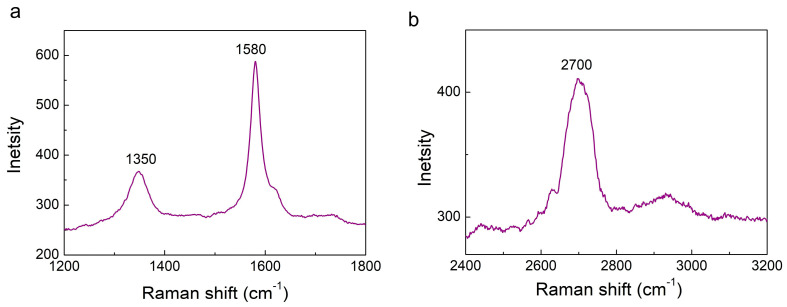
The Raman (**a**) D, G, and (**b**) 2D profiles of the milled CBN/PVP nanoparticles.

The characteristic Raman profiles of the ball-milled graphite nanoparticles functionalized with PVP molecules (CBN/PVP) are shown in Figure 1a,b. The presence of a D band at ≈1350 cm^−1^, a G band at ≈1580 cm^−1^, and a 2D band at ≈2700 cm^−1^ can be seen in the graphitic structure. All three bands (D, G and 2D) are visibly broadened. The G peak corresponds to the optical E_2g_ phonons and is associated with the stretching mode of the C–C bond (i.e., sp^2^ bond). The D peak represents the breathing mode of aromatic rings that arise due to defects in the sample. D peaks and their overtone 2D peaks are often used as a measure of the degree of defects in a graphitic structure. Thus, the broadened D and 2D bands can be assigned to the defects and disordering involved in the hexagonal graphitic layers upon milling, as well as the decrease in the lateral dimension of the milled CBN/PVP sample. Additionally, the intensity of the G band is still higher than that of the 2D band, indicating the multilayer graphite nature [20].

#### 3.1.2. XRD Analysis

**Figure 2:**
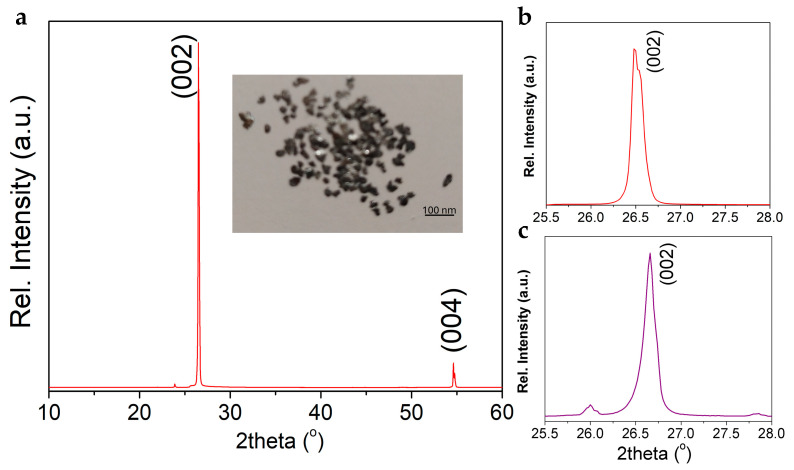
(**a**) XRD pattern and picture of pristine milled CBN/PVP sample and profiles of (002) reflection (**b**) before and (**c**) after milling.

The crystallinity of the pristine graphite used as a starting material can strongly influence the properties of the graphitic material obtained after appropriate processing [20]. The XRD analysis of the pristine, metallic-gray graphite flakes (a photo is shown in Figure 2) revealed the presence of strong and sharp ((002) and (004)) characteristic reflections for S.G. *P* 63 *m c* (No.186). The average coherence length in this direction was found to be (6 ±1) nm. A decrease in the intensity of the graphite 002 reflection is observed in the milled sample (CBN/PVP). The corresponding lattice parameters are *a* = *b* = 2.4613 Å and *c* = 6.7062 Å. The asymmetric shape of the (002) reflection highlights the significant amount of defects (dislocations) in the structure of the milled Gr. It is interesting to note that based on the analysis of the (002) diffraction peak profile, performed using SmartLab Studio software, Rigaku Co. (Tokyo, Japan), no significant change in the line broadening was noticed, i.e., no significant reduction in the multilayer structure of graphite is observed upon milling.

#### 3.1.3. ATR-FTIR Spectroscopy

**Figure 3:**
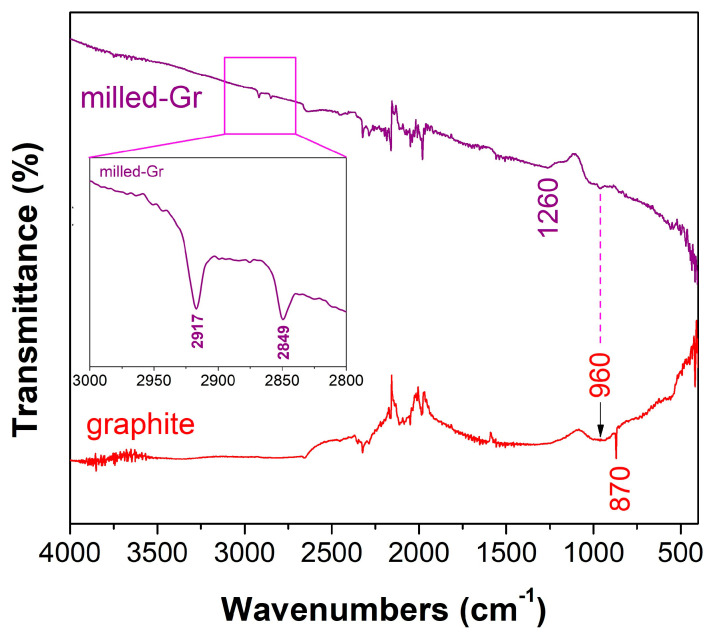
ATR-FTIR spectra of pristine graphite and PVP-functionalized milled graphite (CBN/PVP).

The ATR-FTIR spectra of the pristine graphite flakes (SGL Carbon, Bonn, Germany) before and after the ball-milling process are shown in Figure 3. The almost featureless characteristics of the spectra are due to the metallic-gray (dark-gray) color of the graphite (CBN/PVP). The presence of weak transmittance peaks at 870 cm^−1^ in the pristine graphite can be assigned to the asymmetric ring stretching [21]. This peak is absent in the milled graphite sample, indicating the strong disorder inside the structure of the processed sample. An additional broad peak centered at c.a. ≈960 cm^−1^ observed in the ATR-FTIR spectra of both samples could be assigned to C–O stretching, indicating the presence of oxygen-containing groups on the edges of the structural layers. It is interesting to note the two weak peaks observed at 2849 cm^−1^ and 2917 cm^−1^ in milled CBN/PVP (Figure 3), which correspond to the symmetric and antisymmetric stretching of the methylene group (–CH_2_–). Thus, the surface functionalization of milled graphite nanoparticles with PVP molecules is confirmed by the FTIR spectra (absorption bands at 2840–2940 cm^−1^ appear only in PVP-modified carbon nanomaterials). The disordered structure of the milled graphite is more reactive and capable of adsorbing molecules (e.g., PVP, airborne hydrocarbon) on its surface upon exposure to ambient air [22]. In addition, the weak and broad band at ≈1260 cm^−1^, observed in the processed sample, can be assigned to the stretching vibration of the –C–N– bonds due to the adsorbed PVP molecules. The peaks between 2370 and 1780 cm^−1^ are due to the noise of the FTIR spectra.

#### 3.1.4. CBN/PVP Morphology

**Figure 4:**
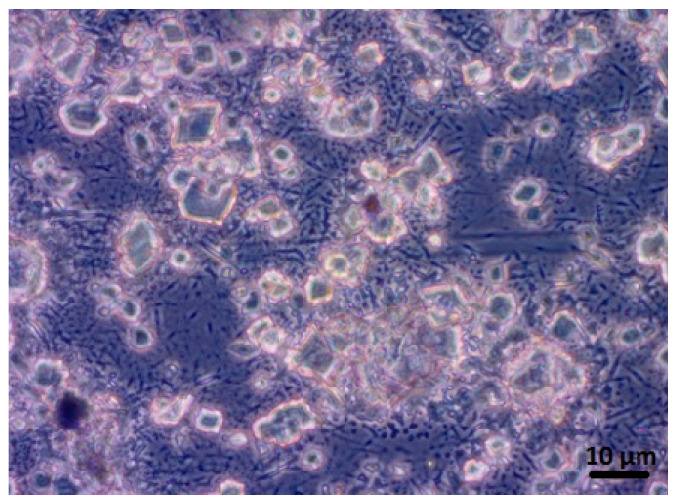
Typical appearance of the carbon-based nanosheets seen on a fluorescent microscope. Magnification 800×.

In the image obtained by fluorescent microscopy (magnification of 800×) shown in Figure 4, the nanosized graphitic structure of CBN/PVP can be observed. The lateral sizes of the graphitic sheets are between 2.5 and 10 µm. The typical irregular shape of the particles can be noticed.

#### 3.1.5. SEM Analysis

**Figure 5:**
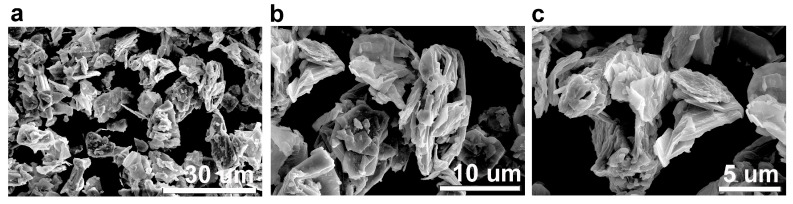
SEM micrographs of the obtained the ball-milled CBN/PVP sample (magnification: (**a**) 2500×, (**b**) 6500× (**c**) 10,000×).

The scanning electron (SEM) micrographs of the graphitic structure, CBN/PVP, obtained after the ball-milling process are shown in Figure 5. The perfect platelet shape of the pristine graphite flakes was destroyed after 20 h of ball milling, resulting in an irregular particle shape with a flower-like morphology, which is a consequence of the agglomeration of graphene sheets [23]. The lateral size of the graphitic sheets is significantly reduced, ranging from 2 to 10 µm. Graphite particles can be observed as separate entities, with their surfaces covered by PVP molecules.

#### 3.1.6. Wettability

**Figure 6:**
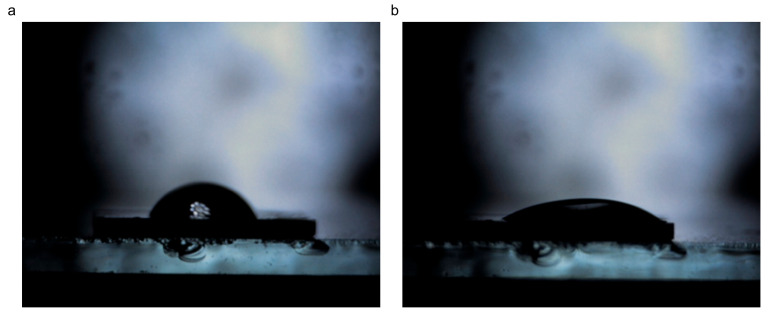
The contact angle between CBN/PVP and water: (**a**) initial—73°, (**b**) after 2 min—29°.

**Figure 7:**
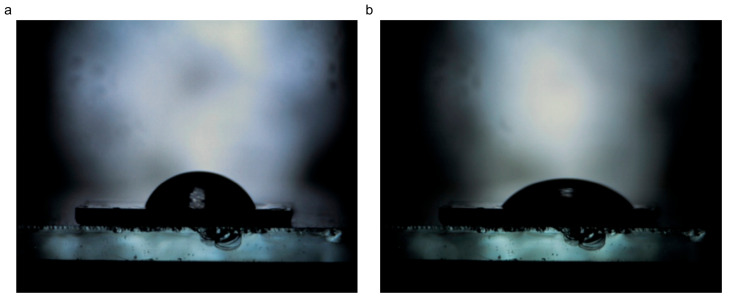
Contact angle between CBN/PVP and ethylene glycol: (**a**) initial—69°, (**b**) after 2 min—47°.

Recent findings have shown that the wetting properties of the graphitic surface are highly influenced by the existence of defects in the structure [24]. Carbon materials with different defect densities can have similar advancing water contact angles (WCAs), but different static contact angles. The hydrophobicity of the graphitic surface is usually due to airborne hydrocarbon contamination [22]. Instead, the intrinsic nature of graphitic carbon is mildly hydrophilic, with a WCA of around 68.6°. The wetting properties are partially dependent on the nature of substrate, too. Figure 6 and Figure 7 depict the water and ethylene glycol droplets on the surface of the milled CBN/PVP sample, respectively. The initial contact angle of deionized water is 73°, while the equilibrium contact angle (obtained after 2 min) is 29° (Figure 6). For ethylene glycol, the initial contact angle is 69° and the equilibrium contact angle (obtained after 2 min) is 47° (Figure 7).

### 3.2. Cytotoxic Effects of CBN/PVP Nanoparticles on Cancer Cells

#### 3.2.1. MTT Assay

**Figure 8:**
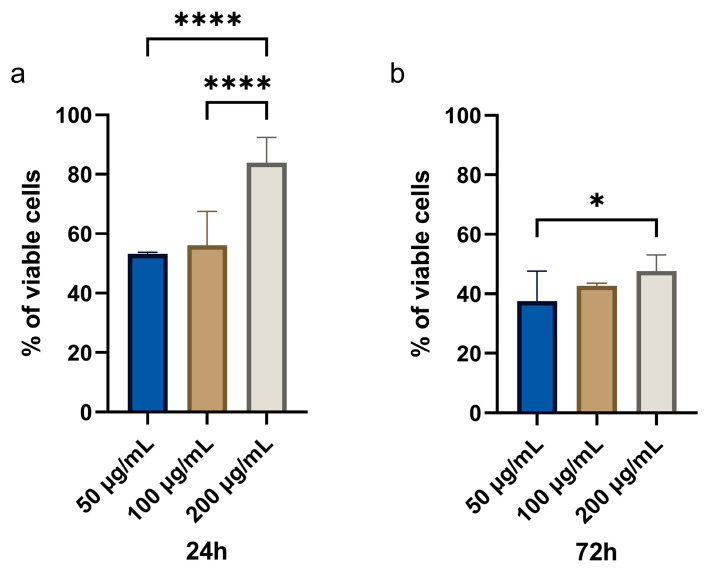
The percentage of viable OSCC cells following treatment with CBN/PVP nanoparticles at concentrations of 50 µg/mL, 100 µg/mL, and 200 µg/mL over two time points: (**a**) 24 h and (**b**) 72 h. Each bar represents the mean ± standard error of the mean (SEM) for the percentage of viable cells under the specified conditions. * *p* < 0.05, **** *p* < 0.0001.

To investigate the cytotoxic effects of the novel material on cancer cells, we applied the MTT test on cells treated with three different concentrations of CBN/PVP nanoparticles with 24 h and 72 h of exposure. Interestingly, lower concentrations of CBN/PVP nanoparticles exerted a more pronounced cytotoxic effect than the highest concentration. Namely, after 24 h (Figure 8), cell viability dropped to approximately 50% in cultures treated with 50 µg/mL and 100 µg/mL, while treatment with 200 µg/mL of G/GO-PVP resulted in 80% cell viability—significantly higher than both the 50 µg/mL and 100 µg/mL groups (*p* < 0.0001). At 72 h, the viability dropped below 40% in cells treated with the lowest concentration of CBN/PVP, and was significantly lower than in cells treated with 200 µg/mL CBN/PVP (*p* = 0.0174) (Figure 8 and Figure S1a).

#### 3.2.2. Gene Expression in OSCC Cells

**Figure 9:**
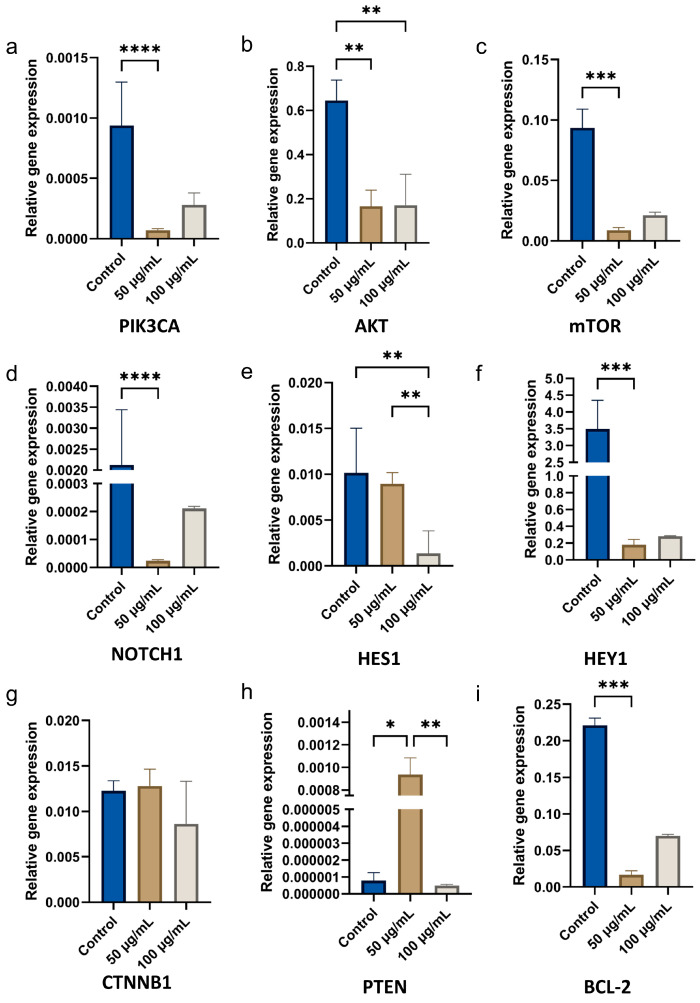
Relative gene expressions of *PI3K*, *AKT*, *mTOR*, *Notch1*, *HES1*, *HEY1*, *CTNNB1*, *PTEN*, and *BCL-2* in OSCC cells treated with CBN/PVP at 50 µg/mL and 100 µg/mL concentration, compared to untreated control cells. Gene expression was assessed in control cells (complete medium without treatment) and in cells treated with two concentrations of CBN/PVP (medium with 50 µg/mL and high 100 µg/mL), with data presented as mean ± SEM. * *p* < 0.05, ** *p* < 0.01, *** *p* < 0.001, and **** *p* < 0.0001.

To determine the possible mechanisms of CBN/PVP action, cells were treated for 72 h with the two lower concentrations that had a greater cytotoxic effect, and the relative expressions of the PI3K/AKT/mTOR (PAM), Notch1/Hes1, and Wnt/β-catenin signaling pathways, along with the anti-apoptotic *BCL-2*, transcription factor *HEY1,* and tumor suppressor *PTEN* genes were examined (Figure 9). It appeared that CBN/PVP treatment with the 50 µg/mL concentration significantly suppressed the expression of *PIK3CA* (*p* < 0.0001), AKT (*p* = 0.0005), mTOR (*p* < 0.0001), *NOTCH1* (*p* < 0.0001), *HES1* (*p* = 0.002), *HEY1* (*p* = 0.0002), and *BCL-2* (*p* < 0.0001), while β-catenin (*CTNNB1* gene) expression remained unaffected. This material showed an effect on *PTEN*, leading to a significant activation of this tumor suppressor (*p* = 0.0179).

#### 3.2.3. Effects of CBN/PVP Nanoparticles on Cell Cycle and Invasion of Oral Cancer Cells

**Figure 10:**
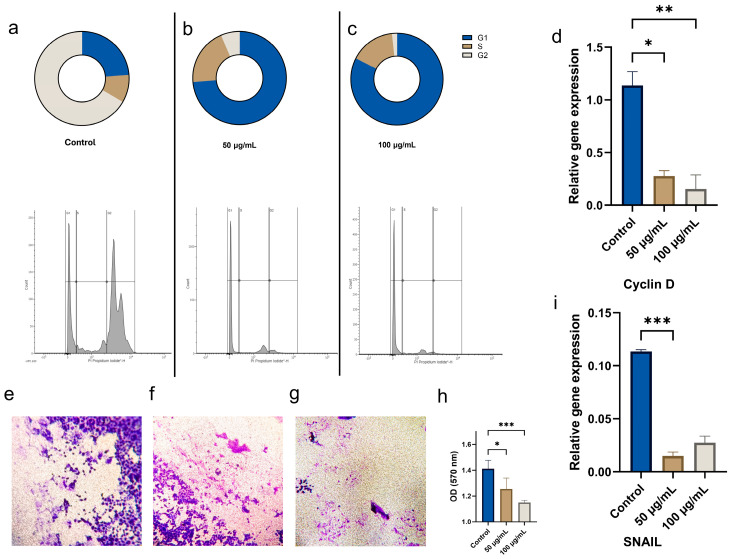
Effect of CBN/PVP on cell-cycle phase distribution, gene expression, and cell invasion. Cell-cycle phase distribution in (**a**) untreated cells (control), and cells exposed to CBN/PVP at concentrations of (**b**) 50 µg/mL and (**c**) 100 µg/mL, (**d**) the expression of cell-cycle-related gene Cyclin D, (**e**–**g**) cell invasion (magnification 10×), (**h**) the quantification of invasion, and (**i**) the expression of the related gene *SNAIL* in comparison to untreated control cells. Data are presented as mean ± SEM * *p* < 0.05, ** *p* < 0.01, and *** *p* < 0.001.

Furthermore, our goal was to examine how CBN/PVP affects the cell cycle. The results suggest that it induces cell-cycle arrest in the G1 phase, as evidenced by an increased percentage of OSCC cells in G1 and a corresponding decrease in the S and G2 phases (Figure 10a–c). We also investigated whether CBN/PVP influences *Cyclin D1*, which is involved in cell-cycle regulation. Our results showed a statistically significant decrease in *Cyclin D1* in cells treated with CBN/PVP (*p* < 0.01 for 50 µg/mL and *p* < 0.001 for 100 µg/mL) (Figure 10d).

The invasion assay indicates that CBN/PVP nanoparticles also affect the invasion of cancer cells (Figure 10e–g). The quantification of invasion (Figure 10h) demonstrates a significant reduction in cell migration at both CBN/PVP concentrations, with a marked decrease in the optical density (OD) values at 570 nm compared to the control (*p* < 0.05 for 50 µg/mL and *p* < 0.001 for 100 µg/mL), indicating that CBN/PVP nanoparticles inhibit cell migration in a dose-dependent manner.

There was also a significant downregulation of *SNAIL*, a gene involved in epithelial to mesenchymal transition (EMT) and cell migration, in the CBN/PVP 50 µg/mL group compared to the control (*p* < 0.001), further supporting the inhibitory effect of CBN/PVP nanoparticles on invasion (Figure 10i).

#### 3.2.4. Effects of CBN/PVP Nanoparticles on Reactive Oxygen Species (ROS) Production in OSCC Cells

**Figure 11:**
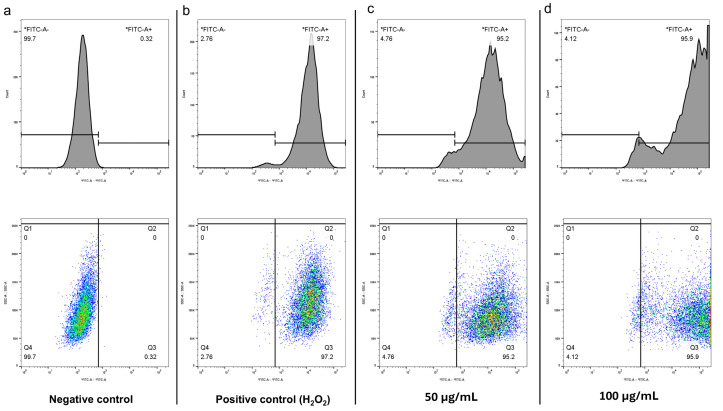
Flow cytometric analysis of ROS production in (**a**) negative control (untreated OSCC cells), (**b**) positive control (cells treated with H_2_O_2_), and (**c**) cells treated with CBN/PVP at 50 µg/mL and (**d**) at 100 µg/mL.

To put forward a possible mechanism of CBN/PVP action, the oxidative damage to OSCC cells via increased ROS production was examined. Flow cytometry experiments with DCFH2-DA fluorescein were used to measure cellular ROS formation and investigate whether differences in intracellular ROS levels appeared in OSCC cells treated with CBN/PVP at different concentrations. ROS predominantly include superoxide anions (O_2_^−^), and to a lesser extent hydrogen peroxide (H_2_O_2_) and hydroxyl radicals (OH). Though less represented, H_2_O_2_ is a stable ROS, able to cross the cell membrane through diffusion mechanisms, and it plays a crucial role in ROS-dependent signaling [25]. Figure 11 depicts the production of ROS in OSCC cells that were not treated with CBN/PVP (Figure 11a), in cells treated with H_2_O_2_ (Figure 11b), and in cells treated with CBN/PVP at concentrations of 50 µg/mL (Figure 11c) and 100 µg/mL (Figure 11d). Our results indicate that the treatment at both tested concentrations increased ROS production.

#### 3.2.5. Nanoparticle Uptake

**Figure 12:**
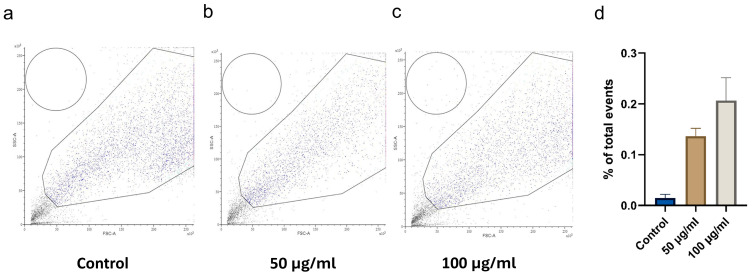
Uptake assay. (**a**–**c**) Cytograms and (**d**) corresponding quantification illustrate the uptake of CBN/PVP nanoparticles by SCC-25 cells at two different concentrations. The data are presented as mean ± SEM.

The cellular uptake of CBN/PVP nanoparticles after 24 h of treatment is quantified and presented in Figure 12. The data show a dose-dependent increase in uptake, though without statistical significance, while the relatively low uptake percentage suggests that the nanoparticles may primarily adhere to the cell membrane or affect the dynamics of cell–nanoparticle interactions.

### 3.3. Effects of CBN/PVP Nanoparticles on DPSC Viability and Osteogenic/Chondrogenic Potential

**Figure 13:**
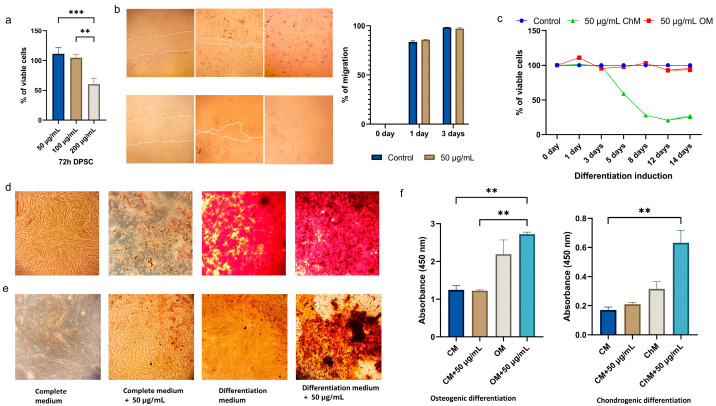
Cell viability and differentiation: (**a**) the percentage of viable DPSCs after 72 h of exposure to CBN/PVP at concentrations of 50 µg/mL, 100 µg/mL, and 200 µg/mL; (**b**) microscopy and measuring of cell migration by wound healing assay (magnification 40×); (**c**) cell viability during differentiation (MTT assay); (**d**) microscopy of osteogenic induction of DPSCs grown in complete medium only, in complete medium supplemented with 50 µg/mL of nanomaterial, differentiation medium alone, and differentiation medium enriched with 50 µg/mL of CBN/PVP (magnification 40×); (**e**) chondrogenic induction of DPSCs grown in complete medium only, in complete medium supplemented with 50 µg/mL of nano-material, differentiation medium alone, and differentiation medium enriched with 50 µg/mL of CBN/PVP (magnification 40×); (**f**) quantification of Alizarin red staining of Ca deposits and quantification of Safranin O staining of lipid droplets. Data are presented as mean ± SEM. ** *p* < 0.01, *** *p* < 0.001.

Finally, we wanted to examine the selectivity of CBN/PVP nanoparticles’ cytotoxicity, and indeed there is a dichotomy of their effects in normal cells versus cancer cells. Namely, the lowest concentration of CBN/PVP (50 µg/mL) showed a stimulative effect on the proliferation of DPSCs, while the highest concentration (200 µg/mL) was cytotoxic; 100 µg/mL had a neither toxic nor stimulative effect (Figure 13a). These results are in complete contrast with the findings in cancer cell cultures, where lower concentrations were cytotoxic, while the highest concentration stimulated cancer cell proliferation.

Adding 50 µg/mL of CBN/PVP into the differentiation medium significantly enhanced osteogenic differentiation (*p* < 0.05) (Figure 13d,f) and chondrogenic differentiation as well (*p* < 0.01) (Figure 13e,f). In summary, these results indicate that the lower concentration of CBN/PVP (50 µg/mL) not only stimulated DPSC proliferation, but also boosted both osteogenic and chondrogenic differentiation. To determine whether the material alone can induce cell differentiation, we also treated the cells with the complete medium supplemented with 50 µg/mL of CBN/PVP for the same duration of 14 days and stained them with Alizarin Red and Safranin. The results indicate that this material cannot independently induce differentiation, but enhances it when present in the appropriate differentiation medium. The results of the MTT assay showed that after the third day, cell viability in the chondrogenic medium with CBN/PVP began to decline sharply. In contrast, in the osteogenic medium with CBN/PVP, the cell number remained stable despite differentiation and, at certain time points, even increased compared to the control (Figure 13c).

## 4. Discussion

In this study, fine graphite platelets were subjected to a ball-milling process. The obtained graphitic nanoparticles subsequently underwent functionalization with PVP molecules, and the material was named CBN/PVP. Based on the Raman spectroscopy and XRD analysis, it was revealed that CBN/PVP possesses a multi-layer structure with high defect density.

The use of nanomaterials, including graphite and graphene derivatives, seems to be a promising approach to solving the severe limitations of conventional cancer chemotherapeutics, such as the insufficient targeting of cancer cells, low solubility and bio-availability, dose-limiting toxicity to healthy tissues, and continually rising rates of drug resistance. Various studies have found that nanoparticles have higher therapeutic efficiency when combined with anticancer drugs [26], including several studies on oral cancer, where the loading of graphene oxide, for instance, with different agents such as saponin [27], doxorubicin [28], and micro-RNA [6], seemed to have satisfying anticancer effects. There are also studies suggesting that carbon-based nanomaterials have anticancer effects by themselves, but the exact mechanisms have not been systematically and comprehensively elucidated [29]. On the other hand, carbon-based nanomaterials are also outstanding in bone regeneration due to their exceptional mechanical strength, biocompatibility, antibacterial properties, and ability to promote cell adhesion and osteogenic differentiation [30,31].

In this study, to the best of our knowledge, PVP-functionalized carbon-based nanoparticles, obtained through the prolonged ball milling of fine graphite platelets (named CBN/PVP), were used for the first time to study their anticancer potential towards OSSC cells and regenerative potential towards dental pulp stem cells.

As already stated, Raman spectroscopy and XRD analysis showed a multi-layered structure of CBN/PVP, with a high defect density; no significant reduction in the multilayer structure of graphite was observed upon milling. The surface functionalization of milled graphite nanoparticles with PVP molecules was confirmed by FTIR spectra (absorption band at 2840–2940 cm^−1^ appears only in PVP-modified carbon nanomaterials), while SEM images showed the irregular shapes of the particles.

Our results show a clear cytotoxic effect of the novel CBN/PVP material on OSSC cells in vitro. Interestingly, the effect was more pronounced at lower concentrations of the nanomaterial than at high concentrations. CBN/PVP induced cell-cycle arrest in the G1 phase, concomitantly with a significant downregulation of *Cyclin D1*, which is known as a positive controller of the cell cycle and is often overexpressed in oral cancer [32]. It has previously been shown that the degradation of Cyclin D1 is sufficient to trigger G1 cell-cycle arrest [33], and our findings corroborate this. The invasion assay indicated that CBN/PVP inhibited oral cancer migration, possibly via the downregulation of *SNAIL*, a transcriptional repressor involved in cell migration and epithelial-to-mesenchymal transition (EMT) in different malignancies, including OSCC [34,35]. These results are in accordance with similar studies which have found that, for instance, graphene oxide by itself, without added chemotherapeutics, exhibits cytotoxic effects on colorectal [36] and hepatocellular carcinoma cells [37], inhibits the migration and invasion of cervical cancer cells [38], and promotes the apoptosis of pheochromocytoma cells [39].

Increased reactive oxygen species (ROS) levels may represent another possible mechanism of CBN/PVP’s anticancer effects. CBN/PVP functions as an electron donor, enhancing ROS production and triggering a mitochondria-dependent apoptotic pathway, while also promoting intracellular ROS generation in a manner dependent on both concentration and exposure time [40]. We showed that CBN/PVP induced oxidative stress, impacting oral cancer cells’ viability. ROS production induces damage to DNA, proteins, lipids, membranes, and organelles, thereby sensitizing cancer cells to apoptosis [41]. In this study, increased ROS levels were linked to a reduction in antiapoptotic BCL-2 expression, which aligns with previous findings that ROS trigger apoptosis by modulating the phosphorylation and ubiquitination of Bcl-2 family proteins, ultimately leading to the downregulation of antiapoptotic proteins [42]. In this study, we also observed remarkable PI3K/AKT/mTOR downregulation. The phosphatidylinositol 3-kinase (PI3K)/AKT/mammalian target of the rapamycin (mTOR) signaling pathway plays a critical role in tumorigenesis, and conversely the inhibition of the PI3K/AKT/mTOR signaling pathway leads to the effective regression of human tumors [43], which is in line with the findings of the present study. Shen et al. also showed that graphene oxide affected the expression of mTOR in the ROS-dependent AMPK/mTOR signaling pathway, leading to the promotion of autophagy and apoptosis in colorectal cancer cells [29]. Dysfunction of the components in the PI3K/AKT/mTOR pathway, such as *PIK3CA* hyperactivity, is also correlated with the loss of *PTEN* function [44]. Our results demonstrated that CBN/PVP activated the tumor suppressor *PTEN*, which could be a potential mechanism for the downregulation of the PI3K/AKT/mTOR signaling pathway. In our study, the CBN/PVP treatment of OSCC cells not only inhibited AKT and mTOR, but also suppressed the NOTCH1 signaling pathway, leading to the downregulation of its target, the *HES1* transcription factor, which plays a crucial role in regulating the cell cycle, proliferation, and survival [45].

One of the most important properties of carbon-based nanomaterials that sets them apart from conventional chemotherapeutics is their ability to target cancer cells not only actively, mostly due to their surface functionalization, but passively as well, as cancer tissues have compromised lymphatic drainage, facilitating the stagnation of nanoparticles within the tumor environment [7].

However, the effects of carbon-based nanomaterials on healthy cells and tissues should also be carefully examined. For instance, nano-graphite induces a stronger toxicological and pro-inflammatory response in murine macrophages compared to carbon nano tubes, likely due to its platelet-like structure and surface characteristics [46]. Li et al. successfully addressed these unfavorable properties of the material through functionalization with PVP, which enhanced immunological biocompatibility, reduced inflammatory cytokine levels, and improved its potential as a drug carrier [47].

The biological characteristics of cancer cells include a larger, deformed nucleus with a perforated envelope, disorganized actin fibers, reduced E-cadherin expression, and increased phosphatidylethanolamine and phosphatidylserine in the outer membrane leaflet, making them more susceptible to graphene’s cytotoxic effects, while healthy cells, with stable membranes, remain unaffected or may even benefit from a regenerative stimulus [48]. Our results show that CBN/PVP does not have the ability to independently initiate osteogenic differentiation, but rather acts as a promoter in the presence of an appropriate induction medium. During differentiation, cells undergo various changes that can also lead to apoptosis [49]. By monitoring cell viability throughout the experiment, we observed that after three days, cell viability in the group with chondrogenic medium sharply declined compared to the control group and the group with osteogenic medium. In contrast, in the osteogenic medium, at a certain point during the experiment, increased proliferation was recorded compared to the control group, which contained only the complete growth medium. These results suggest that the bone environment is a favorable medium in which CBN/PVP can exert a regenerative effect, which requires further investigation [50,51]. One important limitation of the present study is the relatively limited number of tested carbon-based nanomaterial concentrations, i.e., some future studies should definitely examine a wider range of doses applied in cell cultures. The novel material should also be tested on other cell types in order to check its capacity to stimulate bone regeneration. Another limitation of our study is the lack of differentiation confirmation by gene expression analysis of specific markers for osteo-induction (such as *ALP*, *BMP*, *RUNX2*, etc.) and for chondro-induction (such as *COL1*, *COL2A1*, etc.) [52]. Yet, our findings are in accordance with studies that have demonstrated the advantages of different carbon nanomaterial structures in tissue regeneration [10,53].

In other words, CBN/PVP, at an optimal concentration, may exert a dual effect: antineoplastic on tumor cells, and regenerative on healthy cells.

## 5. Conclusions

Polyvinylpyrrolidone (PVP)-functionalized carbon-based nanomaterial (CBN/PVP), obtained through the ball milling of fine graphite flakes, exerted a strong cytotoxic effect on oral cancer cells by promoting oxidative stress and modulating the PI3K/Akt/mTOR pathway. It also caused G1 cell-cycle arrest via Cyclin D1 downregulation and finally inhibited migration by decreasing SNAI1 levels. It is worth noticing that the same concentrations of CBN/PVP nanoparticles had an opposite effect on healthy DPSCs, supporting their survival and boosting their osteogenic and chondrogenic potential. The results of this study suggest that our novel CBN/PVP material may serve as not only an anticancer agent against oral cancer, but aid in tissue regeneration as well.

## Data Availability

The original contributions presented in this study are included in the article/Supplementary Materials. Further inquiries can be directed to the corresponding author.

## Supplementary Materials

The following supporting information can be downloaded at: https://www.mdpi.com/article/10.3390/jfb16030109/s1, Figure S1: Panel plots; Table S1: Primer sequences used in the study.
