# Supplementary material for: A Carbon-Based Nanomaterial with Dichotomous Effects: Antineoplastic on Oral Cancer Cells and Osteoinductive/Chondroinductive on Dental Pulp Stem Cells
Source: J Funct Biomater. 2025 Mar 19;16(3):109. doi: 10.3390/jfb16030109 (PMC11943258; doi:10.3390/jfb16030109)
Supplement: Supplementary file 1 [file jfb-16-00109-s001.zip › jfb-3407511-supplementary.pdf]

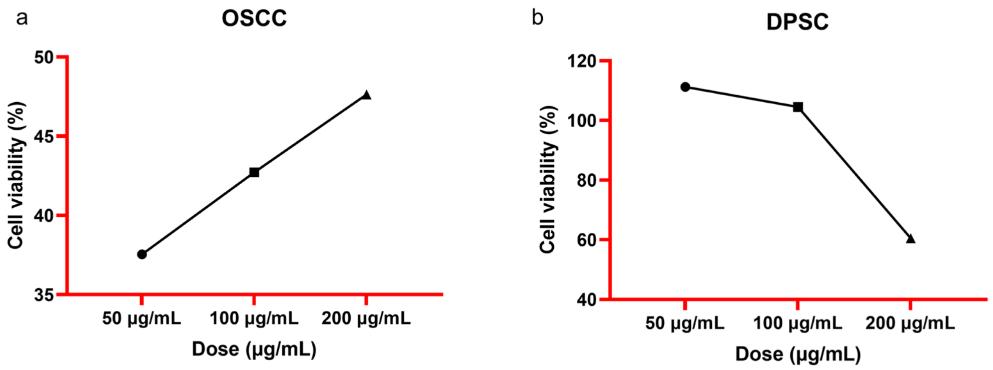

Figure S1. Panel Plots**.** The panel plots compare the dose-response relationship at 72 hours between OSCC (**a**) and DPSC (b). In the OSCC panel, cell viability (%) remains relatively stable with a slight increase as the dose increases from 50 to 200 µg/mL, staying below 50%. In contrast, the DPSC panel shows a significant decrease in cell viability with increasing dose, dropping from 111.2% at 50 µg/mL to 60.8% at 200 µg/mL. This highlights a clear difference in response to treatment between the two cell types.

Table S1. Primer Sequences used in the study.

GENE	DIRECTION	SEQUENCE
Cyclin D1	Rv	5′GGGTGTGCAAGCCAGGTCCA 3′
	Fw	5′ CGGAGGAGAACAAACAGATC 3′
BCL-2	Rv	5’ TGAGCAGAGTCTTCAGAGACAGCC 3’
	Fw	5’ ATGTGTGTGGAGAGCGTCAACC 3’
AKT	Rv	5’GCAGAGAGGTAATCAGCACCAA 3’
	Fw	5’GCAAAGCAGGAGTATAAGAAAGGAA3’
PIK3CA	Rv	5’AAGTGGATGCCCCACAGTTC 3’
	Fw	5’ TTACCCTCTTCTGCCGGAGG 3’
HES1	Rv	5’CCGCGAGCTATCTTTCTTCA 3’
	Fw	5’ TCAACACGACACCGGATAAA 3’
NOTCH 1	Rv	5’CCACGAAGAACAGAAGCACA 3’
	Fw	5’AGCCTCAACATCCCCTACAA 3’
HEY1	Rv	5’CTGGGTACCAGCCTTCTCAG 3’
	Fw	5’CGAGGTGGAGAAGGAGAGTG 3’
PTEN	Rv	5’GGGAATAGTTACTCCCTTTTTGTC 3’
	Fw	5’ACCCACCACAGCTAGAACTT 3’
CTNNB1	Rv	5’ GGTAGTGGCACCAGAATGGATT 3’
	Fw	5’ GCTACTCAAGCTGATTTGATGGA 3’
mTOR	Rv	5’ TGGTTTCCTCATTCCGGCTC 3’
	Fw	5’ GCCGCGCGAATATTAAAGGAA 3’
SNAIL	Rv	5’ GGTCGTAGGGCTGCTGGAA 3’

	Fw	5’ ACCACTATGCCGCGCTCTT 3’
**GAPDH**	Rv	5’ CCC TGT TGC TGT AGC CAA ATT CGT 3’
	Fw	5’ TCA TGA CCA CAG TCC ATG CCA TCA 3’
